# Construction and Comprehensive Analysis of a Stratification System Based on *AGTRAP* in Patients with Hepatocellular Carcinoma

**DOI:** 10.1155/2021/6144476

**Published:** 2021-11-17

**Authors:** Li Wang, Wenjun Zhang, Tao Yang, Le He, Yunmei Liao, Jiaxi Lu

**Affiliations:** ^1^Department of Oncology, Chongqing General Hospital, University of Chinese Academy of Science, Chongqing, China; ^2^Key Laboratory for Biorheological Science and Technology of Ministry of Education (Chongqing University), Chongqing University Cancer Hospital, Chongqing, China

## Abstract

**Background:**

With the development of sequencing technology, several signatures have been reported for the prediction of prognosis in patients with hepatocellular carcinoma (HCC). However, the above signatures are characterized by cumbersome application. Therefore, the study is aimed at screening out a robust stratification system based on only one gene to guide treatment.

**Methods:**

Firstly, we used the limma package for performing differential expression analysis on 374 HCC samples, followed by Cox regression analysis on overall survival (OS) and disease-free interval (PFI). Subsequently, hub prognostic genes were found at the intersection of the above three groups. In addition, the topological degree inside the PPI network was used to screen for a unique hub gene. The rms package was used to construct two visual stratification systems for OS and PFI, and Kaplan-Meier analysis was utilized to investigate survival differences in clinical subgroups. The ssGSEA algorithm was then used to reveal the relationship between the hub gene and immune cells, immunological function, and checkpoints. In addition, we also used function annotation to explore into putative biological functions. Finally, for preliminary validation, the hub gene was knocked down in the HCC cell line.

**Results:**

We discovered 6 prognostic genes (*SKA1*, *CDC20*, *AGTRAP*, *BIRC5*, *NEIL3*, and *CDC25C*) for constructing a PPI network after investigating survival and differential expression genes. According to the topological degree, *AGTRAP* was chosen as the basis for the stratification system, and it was revealed to be a risk factor with an independent prognostic value in Kaplan-Meier analysis and Cox regression analysis (*P* < 0.05). In addition, we constructed two visualized nomograms based on *AGTRAP*. The novel stratification system had a robust predictive value for PFI and OS in ROC analysis and calibration curve (*P* < 0.05). Meanwhile, *AGTRAP* upregulation was associated with T staging, N staging, M staging, pathological stage, grade, and vascular invasion (*P* < 0.05). Notably, *AGTRAP* was overexpressed in tumor tissues in all pancancers with paired samples (*P* < 0.05). Furthermore, *AGTRAP* was associated with immune response and may change immune microenvironment in HCC (*P* < 0.05). Next, gene enrichment analysis suggested that *AGTRAP* may be involved in the biological process, such as cotranslational protein targeting to the membrane. Finally, we identified the oncogenic effect of *AGTRAP* by qRT-PCR, colony formation, western blot, and CCK-8 assay (*P* < 0.05).

**Conclusion:**

We provided robust evidences that a stratification system based on *AGTRAP* can guide survival prediction for HCC patients.

## 1. Introduction

Globally, primary liver cancer is one of the most common cancers, with a high mortality. Hepatocellular carcinoma (HCC) accounts for the great majority of liver cancer, which is genetically defined as a malignancy with unique molecular events and is anticipated to be with high heterogeneity [1]. Despite the widespread application of pathological staging, it still has limits in predicting survival [2]. As a result, a novel stratification system must be developed that provides for precise clinical intervention.

AT1R-associated protein (*ATRAP*) is a molecule specifically interacting with the carboxyl-terminal domain of the angiotensin II (Ang II) type 1 receptor (*AT1R*) [3]. Although *AGTRAP* (*ATRAP*) is found in a variety of human tissues, little is known about it in tumor tissue [4]. Because *AT1R* is involved in the pathophysiology of hypertension, fundamental research has focused on the function of *AT1R* and *ATRAP* in hypertension development [5, 6]. A recent study on illnesses like hypertension and nephrotic syndrome has focused on *AGTRAP*. *ATRAP* expression was positively associated with *AT1R* gene expression in 22 kidney biopsy specimens of IgA nephropathy [7]. Furthermore, *ATRAP* expression of hypertension patients was significantly lower than that of normotensive patients in 36 visceral adipose tissues from abdominal surgery [4]. In particular, *ATRAP* expression was strongly associated with inflammatory indicators such as granulocyte and monocyte counts in outpatients with noncommunicable illnesses [8]. Hence, therefore, in order to fill the gap of *AGTRAP* in tumorigenesis, the role of *AGTRAP* in HCC was explored in depth using bioinformatics analysis and assays.

Several signatures have been reported in the previous references for the prediction of prognosis in patients with HCC [9–11]. However, the above signatures are characterized by excessive factors and cumbersome application in clinical settings. Therefore, the study is aimed at screening out a robust stratification system based on only one gene to facilitate prognosis prediction of HCC patients.

## 2. Materials and Methods

### 2.1. Differential Expression Analysis

We download RNA-sequence data (HTseq-FPKM) from the Pancancer Project in The Cancer Genome Atlas (TCGA) database. The expression of *AGTRAP* was compared between the normal and tumor tissues after log2 transformation. In addition, the RNA-sequence data of TCGA-LIHC in the same format was downloaded to perform *AGTRAP* differential analysis of paired (50 pairs) and unpaired (374 HCC tissues and 50 normal adjacent tissue). The thresholds were set to log2(FC) ≥ 4, *P* < 0.05, in the limma package in R software, and the gene set of differential expression was identified.

### 2.2. Construction of PPI Network and Screening of Prognostic Risk Factors

Prognostic risk factors in HCC patients were identified by univariate Cox regression analysis, and thresholds were set to HR > 1, *P* < 0.001. According to Cox regression analysis, gene sets related to overall survival (OS) and progress-free interval (PFI) were identified. Then, we intersected the above three gene sets and screened the hub genes involved in the construction of a protein-protein interaction (PPI) network. The PPI network was constructed by using the STRING tool and Cytoscape software. We retained only a gene with top topological degree.

### 2.3. Clinical Prognosis Analysis and Construction of Stratification System

The clinical data was downloaded from the TCGA database, including age, gender, pathological stage, T staging, N staging, M staging, grade, residual size, AFP, albumin, and vascular invasion. We calculated the median expression of *AGTRAP* of HCC patients, which is used to select “high-risk” and “low-risk” groups. Kaplan-Meier survival analysis and log-rank test were used to suggest the survival differences in the two groups. In addition, independent prognostic factors were identified by univariate and multivariate Cox regression analysis. We used the “rms” package in R software to plot a nomogram for visualizing the prognosis value of *AGTRAP*. The distinction and calibration were evaluated by the ROC curve and calibration curve.

### 2.4. Enrichment Analysis of Differentially Expressed Genes

We divided all HCC samples into two groups (*AGTRAP*-high and *AGTRAP*-low) based on the median expression of *AGTRAP*. The differentially expressed genes (DEGs) in *AGTRAP*-high samples and *AGTRAP*-low samples were screened using the limma package in R software. The thresholds were set to ∣log2(FC) | >2 and *P*.adj < 0.05. Moreover, Gene Ontology (GO) and Kyoto Encyclopedia of Genes and Genomes (KEGG) analyses were performed using related packages.

### 2.5. Immune-Infiltration Analysis

The immune-infiltration algorithm used in this study was ssGSEA, which was implemented through the GSVA package in R software. The correlation between *AGTRAP* and immune function, immune cells, and immune checkpoints was analyzed. Pearson correlation analysis was used to verify the correlation between the risk group and immune cell infiltration.

### 2.6. In Vitro Assays

In this study, we used cell culture, transfection, CCK-8, and qRT-PCR as in vitro assays. The Shanghai Cell Institute Country Cell Bank provided the normal and HCC cell lines. GenePharma generated and annealed small-interfering RNA (si-RNA-1/2/3) oligos for *AGTRAP* and a general negative control. Following the manufacturer's procedure, each siRNA duplex was transfected into the cells using Lipofectamine® 2000 (Invitrogen, Carlsbad, CA, USA). The antibodies against AGTRAP and GAPDH were all obtained from Abcam. GAPDH served as the internal control. The sequences of the primers used for qRT-PCR are as follows in reference [12]. The details of the methods are provided in reference [13]. In addition, transwell, clone formation, western blot, and other detailed experimental processes are discussed in our previous study [14].

### 2.7. Statistical Analysis

All statistical analyses were performed using the R software (v.4.0.1). Detailed statistical methods about transcriptome data are covered in the bioinformatics method section. *P* < 0.05 was considered statistically significant.

## 3. Results

### 3.1. Screening of a Hub Prognosis Gene in Patients with Hepatocellular Carcinoma

**Figure 1:**
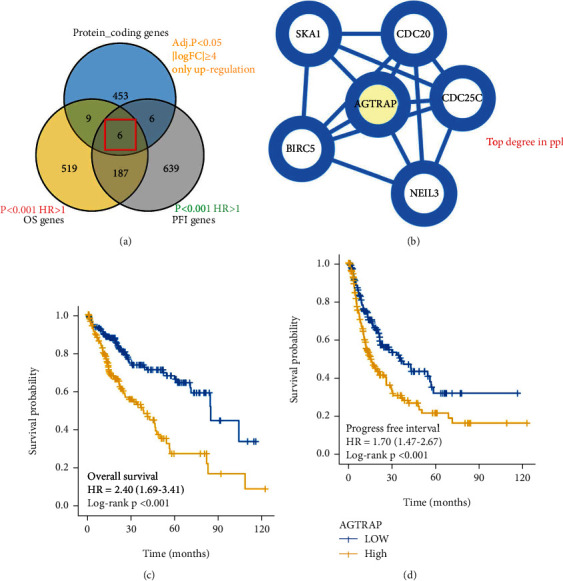
Screening of a hub prognosis gene in patients with HCC: (a) an intersection of protein-coding genes, OS genes, and PFI genes; (b) the PPI network of 6 hub genes for calculating topological degree; (c) Kaplan-Meier survival analysis of *AGTRAP* in OS of patients with HCC; (d) Kaplan-Meier survival analysis of *AGTRAP* in PFI of patients with HCC.

Differential expression analysis was performed on the transcriptome data of all samples in the TCGA-LIHC cohort (50 adjacent normal samples and 374 HCC tissues samples). In order to identify potential protooncogenes, we screened only the upregulated genes in HCC tissues, and finally, 474 hub protein-coding genes were identified. Meanwhile, univariate Cox regression analysis was performed on 374 patients corresponding to transcriptome data. We selected risk factors with HR > 1 for screening and finally identified 721 risk factors related to OS and 838 risk genes related to PFI for HCC. Finally, we intersected the above genes and further screened the 6 hub genes involved in the construction of the PPI network, as shown in Figure 1(a). In the PPI network, we calculated the topological degree for the 6 hub genes and determined *AGTRAP* as the final factor, as shown in Figure 1(b). In addition, our survival analysis for 374 HCC patients also showed significant predictive performance in *AGTRAP* (*P* < 0.001), as shown in Figures 1(c) and 1(d).

### 3.2. Independent Prognostic Role of *AGTRAP* in Progress-Free Interval and Overall Survival

**Figure 2:**
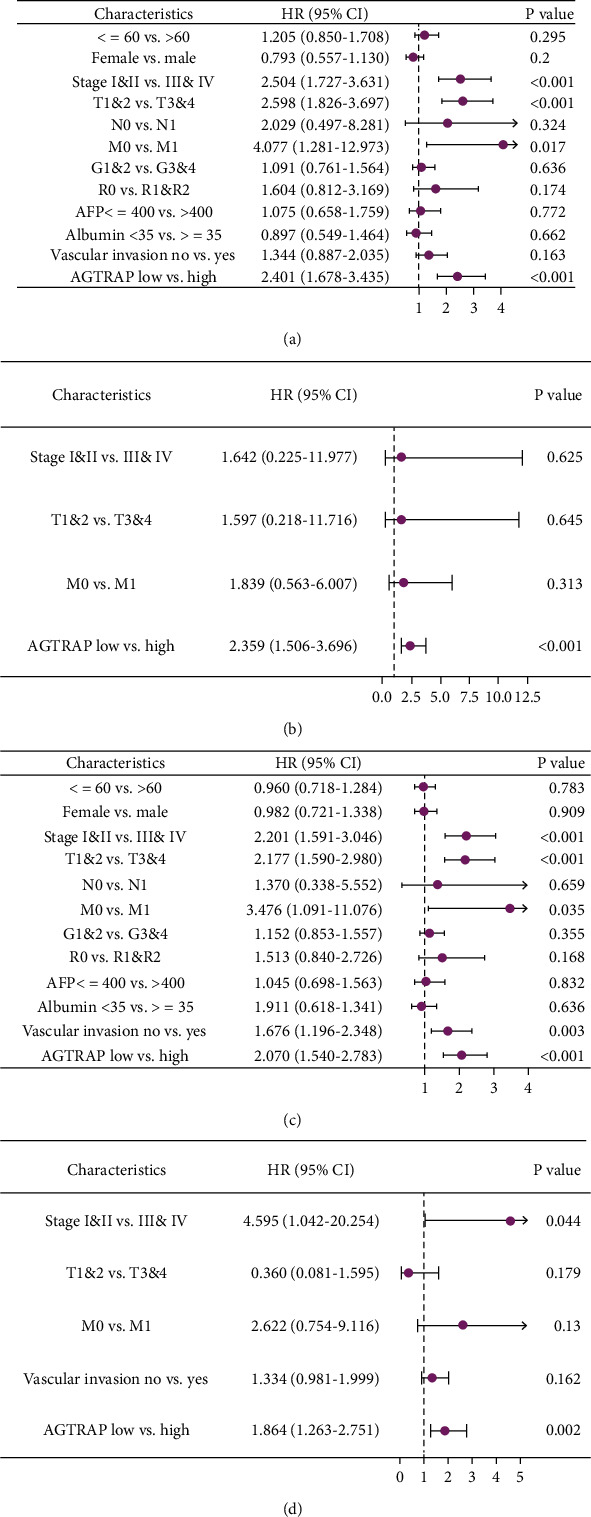
Cox regression analysis of *AGTRAP* in progress-free interval and overall survival: (a) univariate Cox regression analysis based on *AGTRAP* and clinicopathologic factors in OS; (b) multivariate Cox regression analysis based on *AGTRAP* and clinicopathologic factors in OS; (c) univariate Cox regression analysis based on *AGTRAP* and clinicopathologic factors in PFI; (d) multivariate Cox regression analysis based on *AGTRAP* and clinicopathologic factors in PFI.

In order to further explore the independent prognostic ability of *AGTRAP* with clinicopathologic factors, we again conducted univariate and multivariate Cox regression analysis in 374 HCC patients. In OS, univariate Cox analysis revealed that *AGTRAP* with the same pathological staging and T staging were risk factors (*P* < 0.05), as shown in Figure 2(a). Moreover, further multivariate Cox analysis showed that only *AGTRAP* was independently associated with OS (*P* < 0.05), as shown in Figure 2(b). In PFI, similar to the results of Cox analysis in OS, *AGTRAP* was also a high-risk factor for HCC recurrence (*P* < 0.05), as shown in Figures 2(c) and 2(d). Taken together, the results may imply that *AGTRAP* may be an independent prognostic predictor for HCC patients.

### 3.3. Construction and Validation of Visual Prognostic Stratification System

**Figure 3:**
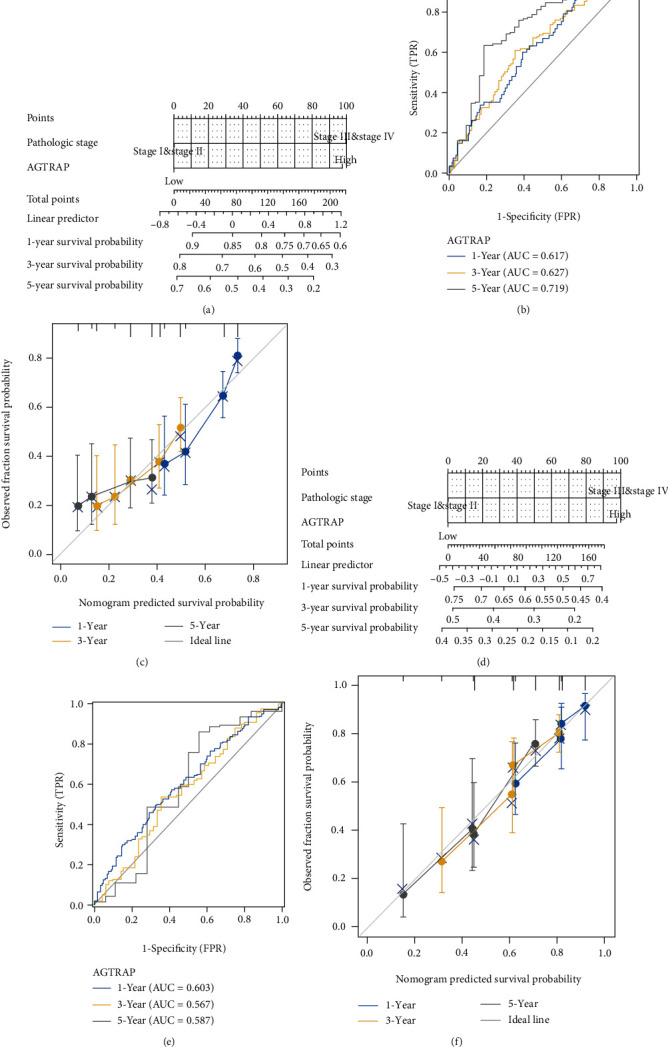
Construction and validation of visual prognostic stratification system: (a) nomogram in OS of HCC patients; (b) ROC analysis for predicting 1.3- and 5-year survival probabilities; (c) calibration analysis for predicting 1.3- and 5-year survival probabilities; (d) nomogram in PFI of HCC patients; (e) ROC analysis for predicting 1.3- and 5-year recurrence probabilities; (f) calibration analysis for predicting 1.3- and 5-year recurrence probabilities.

Considering the clinical value of the pathological stage, we combined the pathological stage and the significance of *AGTRAP* in multivariate Cox analysis to construct two visual stratification systems for OS and PFI, as shown in Figures 3(a) and 3(d). It is worth mentioning that ROC analysis and calibration curve also showed that the stratification system has good predictive value, as shown in Figures 3(b), 3(c), 3(e), and 3(f). The AUC values of 1-, 3-, and 5-year OS are 0.617, 0.627, and 0.719, respectively. Similarly, the AUC values of 1, 3, and 5 years in PFI are 0.603, 0.567, and 0.587, respectively.

### 3.4. Expression Landscape and Clinical Correlation Analysis of *AGTRAP*

**Figure 4:**
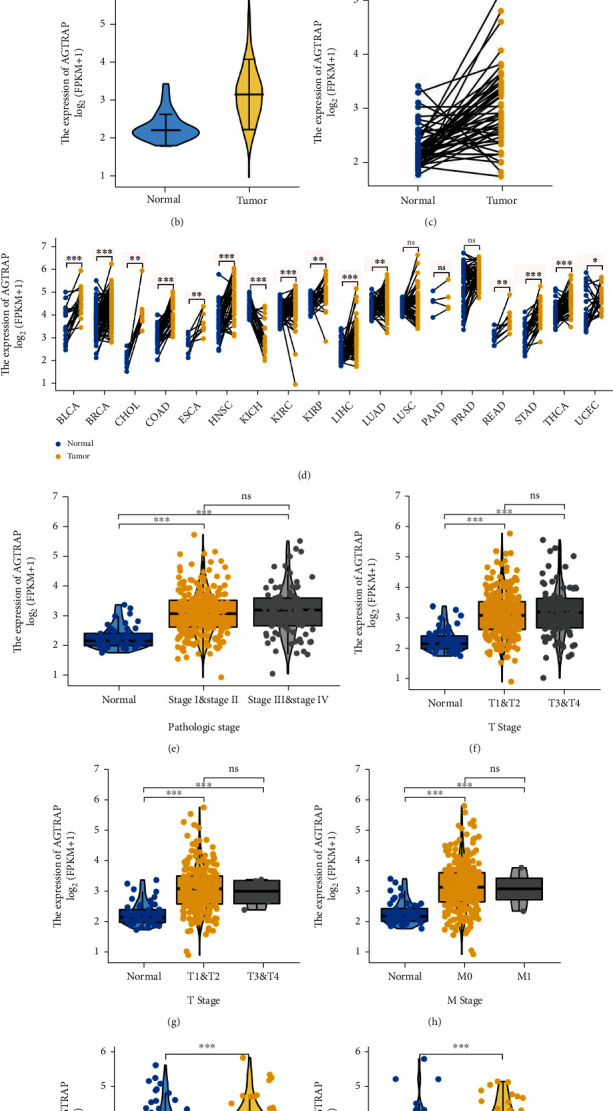
Expression landscape and clinical correlation analysis of *AGTRAP*. (a) Differential unpaired expression of *AGTRAP* in pancancer patients. (b) The unpaired expression of *AGTRAP* was explored in TCGA-LIHC dataset. (c) The paired expression of *AGTRAP* was explored in TCGA-LIHC dataset. (d) Differential paired expression of *AGTRAP* in pancaner patients. Clinical correlation analysis of *AGTRAP* with (e) pathological stage, (f) T staging, (g) N staging, (h) M staging, (i) grade, and (j) vascular invasion.

To further explore the expression of the stratification system in pancancers, we downloaded expression profile raw data of pancancers from the TCGA database. The results showed that *AGTRAP* was upregulated in most tumors in the unpaired differential analysis, as shown in Figure 4(a). In particular, *AGTRAP* was overexpressed in all tumor tissues in the paired differential analysis, as shown in Figure 4(d). In detail, compared with normal liver tissues, *AGTRAP* was overexpressed in HCC tissues (Figures 4(b) and 4(c)). As illustrated in Figures 4(e)–4(j), for the significant differential clinicopathologic factors, *AGTRAP* was overexpressed in T staging, N staging, M staging, pathological stage, vascular infiltration, and G3-G4 (*P* < 0.05).

### 3.5. Potential Prognostic Significance of *AGTRAP* in Clinical Subgroups

**Figure 5:**
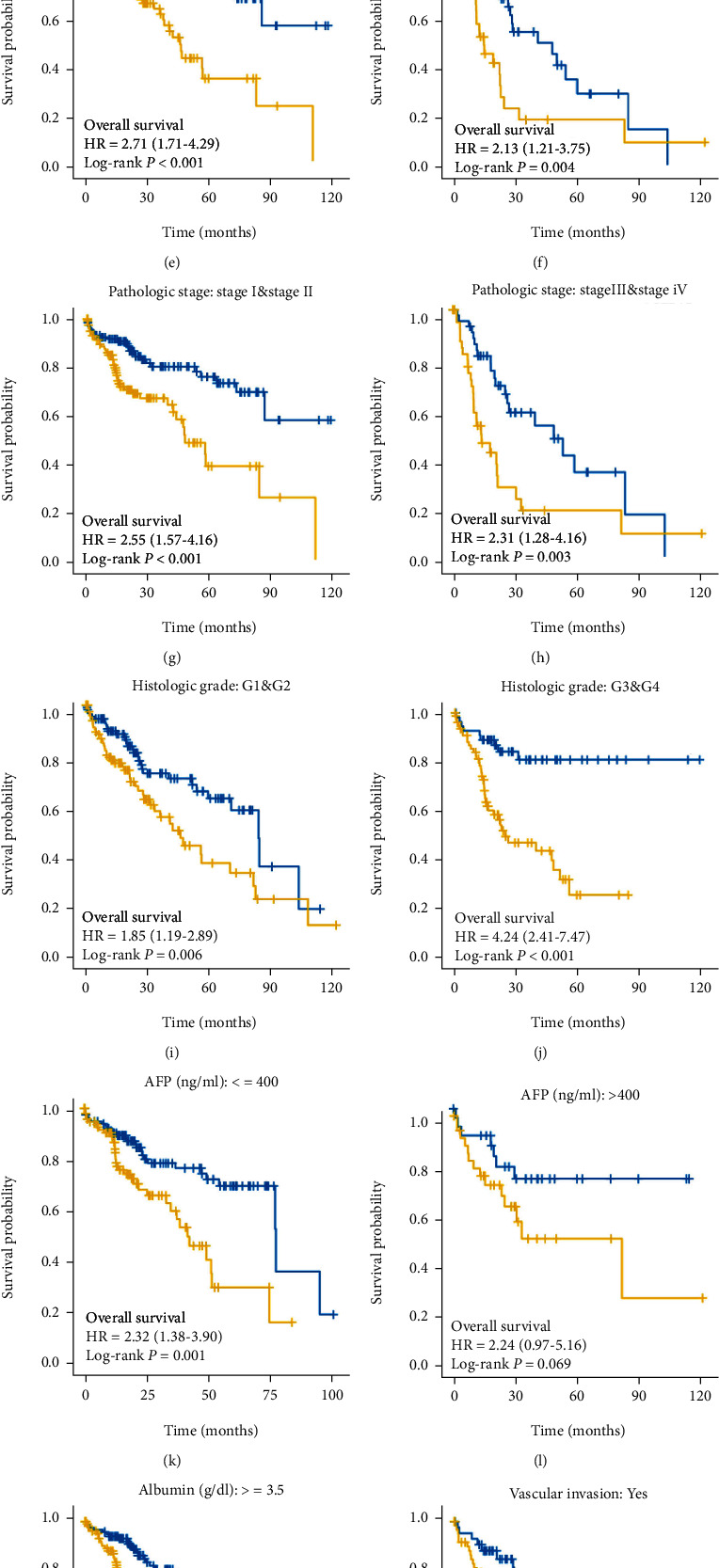
Kaplan-Meier survival analysis in clinical subgroups. Kaplan-Meier survival analysis of subgroups, including (a, b) age, (c, d) gender, (e, f) T staging, (g, h) pathological stage, (i, j) grade, (k, l) AFP, (m) albumin ≥ 3.5 g/dl, and (n, o) vascular invasion.

The stratification system has been demonstrated to have excellent risk stratification value in HCC patients from the TCGA cohort. Subsequently, we performed survival analysis for different clinical subgroups, as shown in Figures 5(a)–5(o). It is worth mentioning that since the number of patients with N1, M1, and R1/R2 is less than 10, we did not perform subgroup survival analysis for N staging, M staging, and residual size. Our results showed that in different subgroups of the Kaplan-Meier analysis, the overall survival time of HCC patients with high expression of *AGTRAP* was significantly shorter than that with low expression of *AGTRAP* (*P* < 0.05).

### 3.6. Analysis of Potential Biological Mechanisms Involving *AGTRAP*

**Figure 6:**
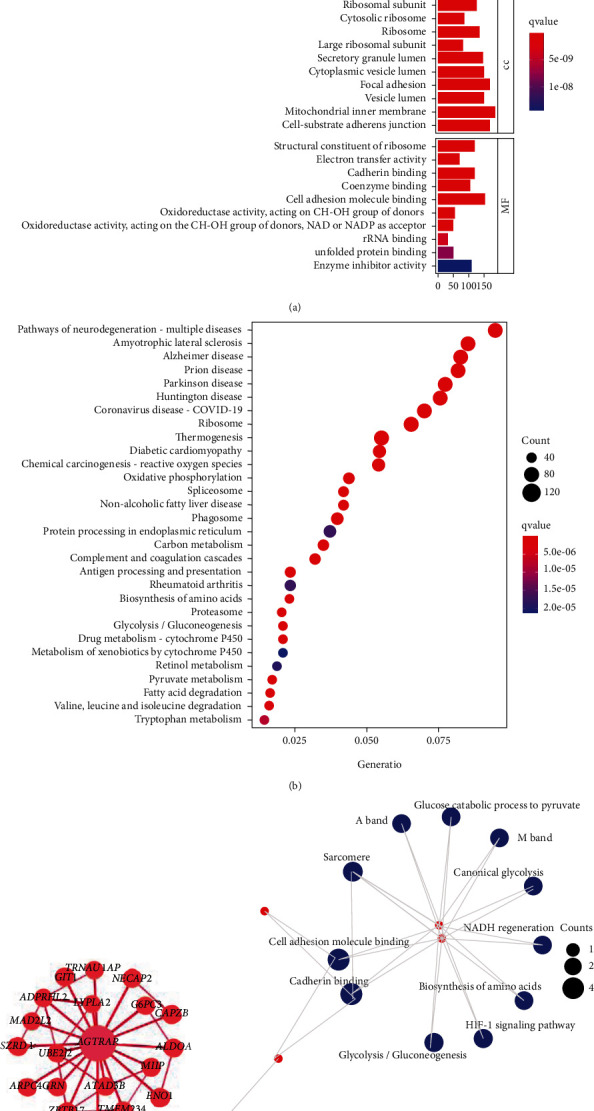
GO and KEGG enrichment analysis of differential genes distinguished by *AGTRAP* expression: (a) GO enrichment analysis of BP, CC, and MF; (b) KEGG enrichment analysis; (c) coexpression network analysis; (d) pathways in which coexpressed genes may be involved.

To further explore the biological mechanisms of *AGTRAP*, we calculated the median expression of *AGTRAP* in HCC patients, which is used to select the “high-risk” and “low-risk” groups. We used the limma package in R software to explore the differential expression of genes in the two groups. According to the threshold of the method section, we screened a total of 2467 upregulated genes and 711 downregulated genes, which may be involved in the regulation with *AGTRAP*. KEGG and GO enrichment analyses were performed for the above genes, as shown in Figures 6(a) and 6(b). GO enrichment analysis showed that *AGTRAP* and its coexpressed genes may be involved in cotranslational protein targeting to the membrane, RNA catabolic process, mRNA catabolic process, etc. KEGG analysis revealed that the above genes may be involved in pathways of neurodegeneration, Alzheimer disease, amyotrophic lateral sclerosis, and other autoimmune pathways. On the basis of differential genes, we analyzed the coexpression gene network of AGTRAP and conducted gene enrichment analysis again. Only 20 genes are coexpressed with AGTRAP, including NECAP2, GP6C3, CAPZB, ALDOA, MIIP, ENO1, ATAD3B, TMEM234, UBE2M, ATAD3B, 2BTB17, 4GRN, ARPC, UBE2J2, SZED1, MAD2L2, ADPRHL2, GIT1, TRNAU1AP, and LYPLA2, as shown in Figure 6(c). Interestingly, these genes may be involved in the HIF-1 signaling pathway, glycolysis, and other biological processes, as shown in Figure 6(d).

### 3.7. A Comprehensive Analysis of Immune Function Based on the Stratification System

**Figure 7:**
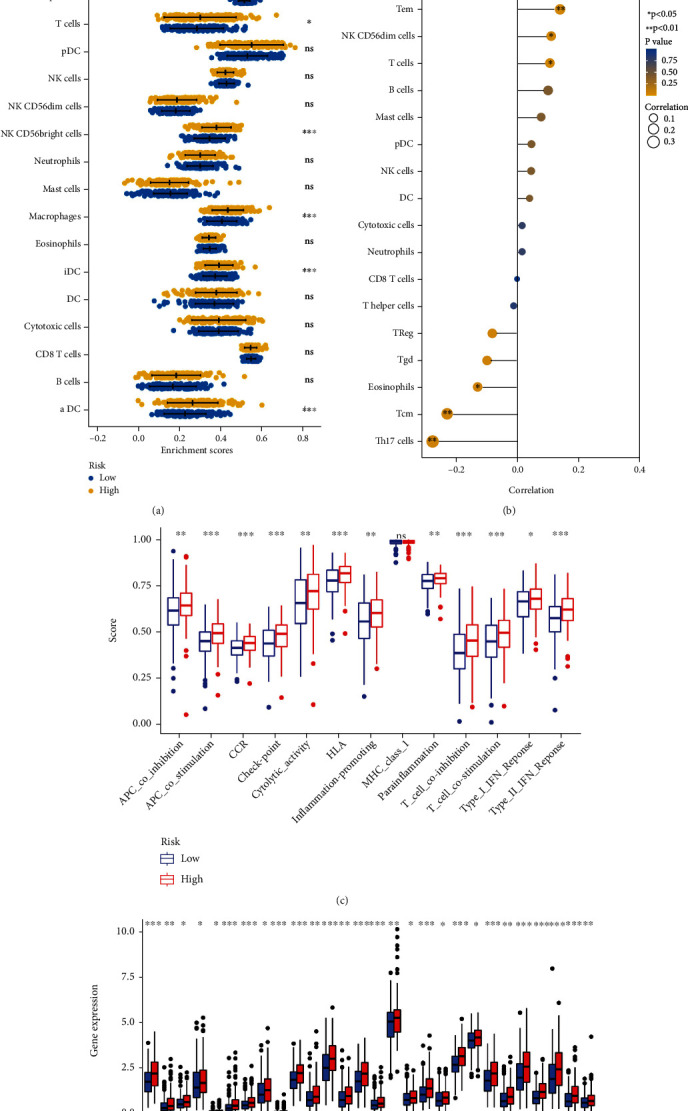
Comprehensive immunological analysis in patients with different risks: (a) differential expression analysis of 24 immune cells; (b) Pearson analysis of 24 immune cells; (c) differences in immune function; (d) mRNA expression of immune checkpoints in patients at different risks. ^∗^*P* < 0.05, ^∗∗^*P* < 0.01, and ^∗∗∗^*P* < 0.001.

Metabolic reprogramming and immune escape are independent predictors of patient survival, and changes in the immune microenvironment regulate tumor progression [15]. Therefore, we used the ssGSEA algorithm to comprehensively analyze the immune cell content, immune cell correlation, immune function, and immune checkpoint in liver cancer tissue. HCC patients were divided into a high-risk group and a low-risk group. It showed that Th2 cells, Th17 cells, Th1 cells, TFH, Tcm, T cells, NK CD56^bright^ cells, macrophages, iDC, and aDC differed significantly between the high-risk and low-risk groups (Figure 7(a)). Moreover, the results showed that *AGTRAP* was negatively correlated with only 3 immune cells, including eosinophils, Tcm, and Th17 cells, and 10 immune cells were positively correlated (Figure 7(b)). Interestingly, analysis of immunologic function confirmed significant differences between the low- and high-risk groups for other immunological functions except MHC class I (*P* > 0.05), as shown in Figure 7(c). Finally, it is worth noting that given the importance of checkpoint immunotherapy, all have significant differences in the expression of immune checkpoints between different risk groups, as shown in Figure 7(d).

### 3.8. In Vitro Assays for Validation

**Figure 8:**
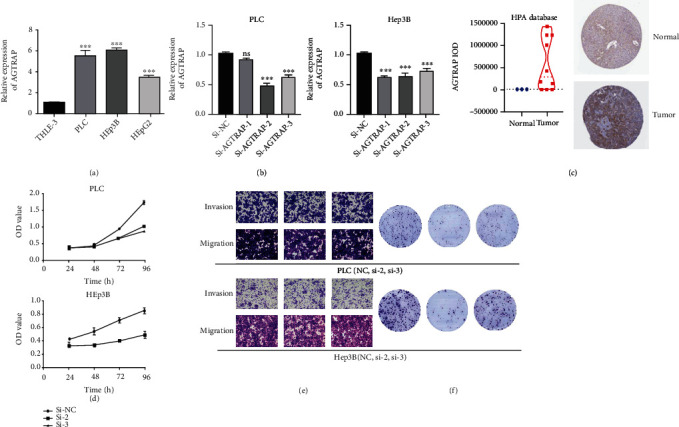
Cell function assays of knockdown *AGTRAP*: (a) relative expression of *AGTRAP* mRNA in cell lines; (b) relative expression of *AGTRAP* mRNA in HCC cell lines transfected with si-*AGTRAP*; (c) IOD value analysis of IHC in the HPA database; (d) CCK-8 assays in HCC cell lines transfected with si-*AGTRAP*-2/3; (e) transwell assays in HCC cell lines transfected with si-*AGTRAP*-2/3; (f) clone formation assay in HCC cell lines transfected with si-*AGTRAP*-2/3. ^∗^*P* < 0.05, ^∗∗^*P* < 0.01, and ^∗∗∗^*P* < 0.001.

To further validate the above bioinformatics results, we detected the expression level of *AGTRAP* mRNA in HCC cell lines. The results showed that expression of *AGTRAP* was upregulated in HCC cell lines (PLC, HEp3B, and HEpG2) compared to THLE-3, as shown in Figure 8(a). In addition, si-*AGTRAP* and si-NC were transfected in PLC and HEp3B cells, respectively, and qRT-PCR and western blot were used to detect the protein expression of *AGTRAP*. It was found that *AGTRAP* expression was downregulated in HCC cell lines with transfection, as shown in Figure 8(b). Similarly, CCK-8 assays showed that HCC cell proliferation was inhibited after transfection with *AGTRAP*, as shown in Figure 8(d). We calculated the IOD value of IHC for 13 clinical samples in the HPA database about the *AGTRAP* protein expression, as shown in Figure 8(c). We found that the *AGTRAP* protein level is also overexpressed in tumor samples. In the cloning formation assay, we also found that knocking down *AGTRAP* could affect proliferation of HCC cells, as shown in Figure 8(f). Unfortunately, the downexpression of the *AGTRAP* may have no effect on the invasion and migration of HCC cells, as shown in Figure 8(e).

## 4. Discussion

Currently, although the TNM system is used to roughly determine the prognosis of HCC patients [1], different stratification systems are not always effective in predicting prognosis depending on the different genetic characteristics. The risk signatures in the previous references are too complicated to be used by clinicians and are expensive to use. Furthermore, as the largest immune organ, the liver plays an important role in the immune response [16]. Therefore, the study focused on the stratified prognostic value of *AGTRAP* and its impact on the immune microenvironment.

Targeted therapy has been intensively studied in a variety of tumors and has now been shown to be a possible new therapeutic approach [17]. However, compared to other targets, there has been relatively little research on *AGTRAP* and cancer, particularly with regard to its specific mechanisms in HCC. In this study, we performed a comprehensive analysis of *AGTRAP*A in HCC. We compared the genetic landscape of *AGTRAP* across TCGA databases; next, we found a robust correlation between *AGTRAP* and clinicopathologic factors. At the same time, the remarkably prognostic predictive value of *AGTRAP* was through a series of studies. In addition, we further explored the potential mechanism of *AGTRAP* and the impact on immune function. Finally, we further explored the role of *AGTRAP* in the HCC cell line by qRT-PCR and western blot.

However, there are a number of limitations to our study that need to be considered with caution. Our study is based on the TCGA database alone, with no validation of an external dataset and clinical samples. Finally, more functional assays are needed to confirm our findings and to better understand the role of *AGTRAP* in HCC.

## 5. Conclusions

We provide strong evidence that a stratification system based on *AGTRAP* can guide survival prediction in HCC patients and may have an impact on the immune microenvironment in HCC tissues.

## Data Availability

The following information was supplied regarding data availability: data is available at the TCGA (https://portal.gdc.cancer.gov/).
